# A Narrative Systematic Literature Review: A Focus on Qualitative Studies on HIV and Medication-Assisted Therapy in the United States

**DOI:** 10.3390/pharmacy9010067

**Published:** 2021-03-23

**Authors:** Alina Cernasev, Sunitha Kodidela, Michael P. Veve, Theodore Cory, Hilary Jasmin, Santosh Kumar

**Affiliations:** 1 Department of Clinical Pharmacy and Translational Science, University of Tennessee Health Sciences Center, Nashville, TN 37211, USA; 2 Department of Pharmaceutical Sciences, University of Tennessee Health Science Center, Memphis, TN 38163, USA; skodidel@uthsc.edu (S.K.); ksantosh@uthsc.edu (S.K.); 3 Department of Pharmacy Practice, Eugene Applebaum College of Pharmacy and Health Sciences, Wayne State University, 259 Mack Avenue, Detroit, MI 48201, USA; mpveve@wayne.edu; 4 Department of Clinical Pharmacy and Translational Science, University of Tennessee Health Science Center, Memphis, TN 38163, USA; tcory1@uthsc.edu; 5 Health Sciences Library, University of Tennessee Health Science Center, Memphis, TN 38163, USA; hjasmin@uthsc.edu

**Keywords:** HIV positive, persons living with HIV, opioid use disorder, United States

## Abstract

Over the last two decades, the United States (U.S.) has experienced an opioid crisis that has had a significant negative societal and economic impact. Due to the high utilization of opioids in Persons Living with HIV and AIDS (PLWHA), there is a need for a qualitative literature review that presents opioid-use related problems in this population. This study aims to present and identify a thematic overview of the qualitative manuscripts on PLWHA who take opioid medications in the U.S., with a focus on perceptions surrounding medication assisted therapy. The systematic literature search was performed in December 2019. Four databases were searched: PubMed/MEDLINE, Scopus, Web of Science, and Cumulative Index to Nursing & Allied Health Literature (CINAHL). A total of 5348 results were exported from databases into EndNote x9, and duplicates were removed for a total of 3039 unique abstracts to screen. The records were imported into Rayyan, an online platform designed to expedite the screening process. Three authors screened titles and abstracts and determined 19 articles that would be screened in full text. On 9 April 2020, it was determined that eight articles would be included for review. The analysis of the eight manuscripts that fit the inclusion and exclusion criteria revealed barriers and facilitators to medication assisted therapy (MAT) in PLWHA. This review communicates or describes the story of PLWHA who might have delayed access to HIV healthcare providers and the commencement of antiretroviral therapy. In the literature, several studies have focused on the role of physicians in prescribing and addressing the medication regimens but none of the studies examined the role of pharmacists in access to care in this population. Therefore, further research is needed for a better understanding of the social aspects of taking opioid medications in PLWHA and the role of pharmacists within the continuum of care.

## 1. Introduction

Over the last two decades, the United States (U.S.) has experienced an opioid crisis that has had a significant negative societal and economic impact. Prescription opioids are generally used to treat different types of pain, including chronic pain associated with HIV [1,2,3]. Furthermore, people living with HIV/AIDS (PLWHA) are more likely to be prescribed opioids than uninfected individuals [4]. Long-term or overuse of opioids by PLWHA could lead to substance use disorder [2,5,6]. Further, PLWHA with opioid use disorder (OUD) are also at risk for serious opioid-antiretroviral drug interactions and HIV disease progression due to reduced antiretroviral (ARV) drug adherence [4,7,8,9]. While advancements have been made to curtail unnecessary opioid use in the U.S., PLWHA still lacks access to care for treatment of OUD. More data are needed that depict the perspectives of PLWHA with OUD, including barriers and facilitators of both treatments. The present study aimed to examine the literature in this area, given the lack of previous qualitative reviews of PLWHA’s perception of taking opioid medications.

Healthcare professionals have united in their response to the opioid epidemic through several targeted national health initiatives [10,11]. Examples of these initiatives include the implementation of strict community opioid prescriptions surveillance through the prescription drug monitoring program (PDMP), hospital opioid stewardship, and increasing access to naloxone as an over-the-counter product. While considerable progress on decreasing the number of prescribed opioids has been made country-wide, other issues in PLWHA are apparent. The stigma associated with OUD [12,13], access to the medication-assisted treatment (MAT), negative perceptions of loved ones associated with individual who take opioids, and complex drug-drug interactions are often overlooked challenges PLWHA may commonly experience, but are rarely addressed [14]. A systematic literature review demonstrated that the barriers experienced by PLWHA and substance use disorder are interrelated [15]. Further, there is a need not only for the healthcare professional to collaborate but also for integrating services to improve patient outcomes [15]. Further systematic literature reviews are necessary to understand the role of stigma on patients who access MAT programs and are taking methadone.

Previous systematic literature review studies depicted different aspects of opioids, such as side effects, the transmission rate of the virus in non-infected persons, and methadone usage. For example, the authors of a Cochrane study concluded that using oral opioids instead of injectable formulations decreases the drug-related behaviors associated with a high risk of HIV transmission [16]. Furthermore, opioid misuse has been reported to be associated with decreased ARV adherence [17].

There is a lack of previous qualitative systematic reviews in the area of medication adherence to ARV treatment access, the effect of counseling while patients are on ARV medication and opioids, prospective of health care provides who worked with PLWHA who has OUD, and stigma associated with OUD and MAT. Therefore, this systematic exploratory review aims to develop a conceptual map of the existing US qualitative literature, evaluate its quality, and narratively present its results.

## 2. Methods

Systematic literature reviews collect and synthesize all available literature on a given topic. This method provides a space for individual studies to build on each other to influence clinical decision-making, ideally leading to evidence-based outcomes [18]. This systematic review was performed by a pharmacy librarian according to the Preferred Reporting Items for Systematic Reviews and Meta-Analyses (PRISMA) guidelines [19].

Inclusion criteria required qualitative or mixed-method study designs focused on adult PLWHA in the U.S. who received antiretroviral therapy (ART) as well as buprenorphine, methadone, naloxone, or naltrexone for OUD. Studies where pharmacists/physicians were engaged in ARV and OUD medication management were a priority. Studies from 2000 through 2020 were included. Exclusion criteria included any non-English or international studies, as well as any grey literature or quantitative study designs. Studies involving pregnant patients or children were also excluded.

**Figure 1:**
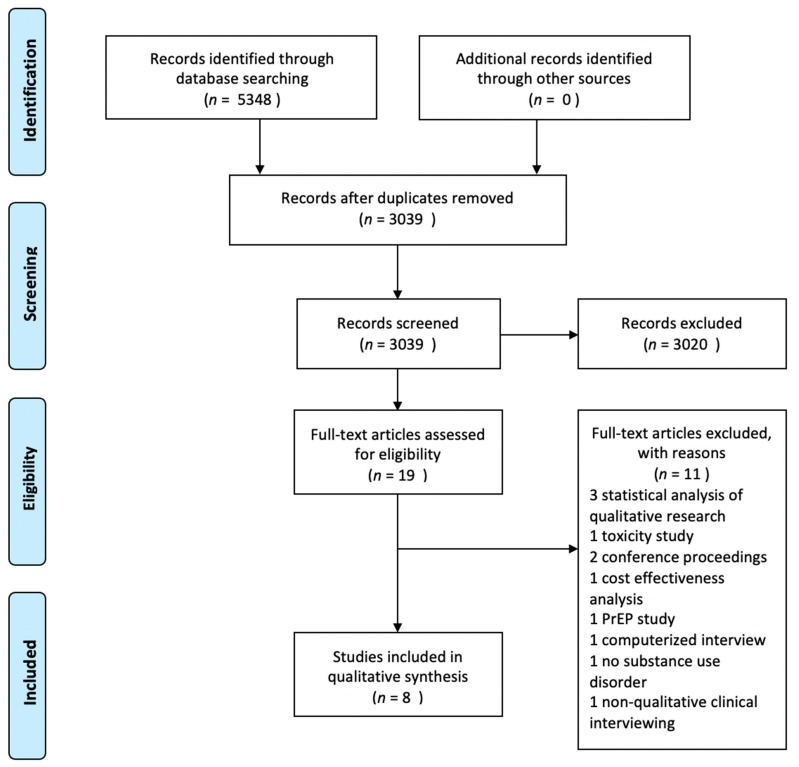
Flow chart based on the PRISMA 2009 diagram.

The systematic literature search was performed in December 2019. Four databases were searched: PubMed/MEDLINE, Scopus, Web of Science, and Cumulative Index to Nursing & Allied Health Literature (CINAHL). Search strategies were created by the pharmacy librarian (Appendix). A total of 5348 results were exported from databases into EndNote x9, and duplicates were removed for a total of 3039 unique abstracts to screen. The unique records were imported into Rayyan (URL https://rayyan.qcri.org/, accessed on 15 December 2019), an online platform designed to expedite the screening process. Three authors screened titles and abstracts and determined that nineteen articles would be screened in full text. On 9 April 2020, it was determined that eight articles would be included for review (Figure 1).

The focus of the current systematic literature review was to include qualitative studies, because contrary to quantitative research, qualitative studies capture in-depth and nuanced data on the social and cultural aspects of HIV, OUD, and access to MAT.

The qualitative study was assessed for risk of bias using the CERQual tool [20], which is presented in Appendix A. The Ottawa New Castle tool could not be used for assessing quantitative studies because only one study used mixed methods. The CERQual tool was used to assess the strength of evidence for qualitative studies [20]. Overall, the majority of qualitative studies were graded as a moderate confidence level, with one study that was graded as low confidence. One factor, which lessened the strength of evidence, was the lack of qualitative studies available to conduct a comparison [20].

## 3. Results

Eight articles were chosen for inclusion in the qualitative narrative review. Out of the eight articles, seven were qualitative and one was a mixed-methods study. Two people abstracted these articles.

A number of studies focus on the barriers and facilitators to MAT access [21,22,23,24,25]. The main conclusion of these articles is that PLWHA face many barriers related to transportation, food security, access to MAT treatment. Other barriers highlighted include obligatory participation in weekly counseling meetings and maintaining treatment agreements. Furthermore, a few studies concluded that having access to and using MAT treatment prescribed by physicians significantly improved the patients′ health and quality of life [23,25].

Using a community-based participatory research approach, Oldfield et al.’s qualitative study was conducted to explore multiple perspectives of HIV care and OUD to develop an instrument to measure quality of integration [21]. Responses revealed patients’ social barriers related to transportation or food security that may be provided through HIV-centered resources; however, patients with OUD alone were unable to obtain similar resources. This was echoed by organization leaders who described PLWHA with or without OUD obtaining more services than those with OUD alone [21]. Participants also recognized that social risks need to be addressed with medical services. Many patients experienced challenges with policies that prevent them from maintaining adherence, and case managers expressed their experience with limited communication between healthcare facilities that put patients at risk [21]. Other patients expressed frustration after being denied same-day entry into buprenorphine or methadone clinics due to waiting lists [21].

Korthuis’ et al. analysis of PLWHA experiences, in quest of buprenorphine maintenance therapy in office-based and opioid treatment program settings, revealed that patients preferred office-based care due to feasibility and accessibility [22]. The authors highlighted several advantages to office-based treatment programs, such as improved medication adherence, the feasibility of making an appointment, and better access for treatment of acute and chronic conditions [22]. This study also discussed the importance of a strong personal connection between patients and providers. Furthermore, the participants in this study preferred to visit an office-based setting because of the benefits that included developing a personal connection with HIV clinic staff, counselors, and providers. This study described the roots of these strong interpersonal connections, including trust, mutual respect, listening, and compassion developed with office-based staff [22].

In the same vein, Eagen et al. explored experiences and perceptions of PLWHA integrated care programs focused on HIV care and addiction treatment. [23] To be included in the study, patients needed to be participating in Health Resources and Services Administration (HRSA)-funded HIV care or buprenorphine/naloxone treatment demonstration projects [23]. The authors found that many participants reported positive experiences and high satisfaction, noting their abilities to return to “normal” life, experiencing more energy, and feeling better than they did on previous methadone treatment [23]. Patients also reported significant improvements in their health and quality of life, allowing them to repair relationships with family members and find enjoyment in daily tasks [23]. Overall, this study reported significant satisfaction with HIV care and buprenorpine/naloxone treatment [23].

Inciardi et al. study focused to obtain a more in-depth understanding of how specific drug-using populations are diverting prescription opioids and other medications or attaining controlled drugs that have already redirected [24]. The study determined that the participants’ abuse of prescription opioids and tranquilizers acted as a gateway, prompting the use of street stimulants, typically methamphetamine or ecstasy. The authors show that the participants had different reasons for using street stimulants, such as obtaining “a better high” when taking club drugs or various other drug combinations [24]. The participants of this study also pointed out their favored drug combinations, such as marijuana, methylphenidate, and alcohol; depressants and/or opioids with methamphetamine; codeine with ecstasy; and hydrocodone with cocaine [24]. Furthermore, the participants in the current study discussed some adherence barriers, including adverse drug reactions to their prescribed ARV regimen. According to the authors, some PLWHA declined to sell medicines. However, there were many participants who sold their medication due to financial hardships [24].

The issues of access and integration of care are discussed through the participant experiences and perspectives within the FAST PATH program [25]. Drainoni et al. point out that although the participants identified numerous positive points regarding the integration of care, they also revealed some obstacles. These barriers include the feasibility of having all their services integrated with-in one location, synchronizing all medical care and substance abuse treatment, and addressing multiple medical and psychosocial issues together [25]. This study also revealed that one of the major obstacles for these participants was the obligatory participation in weekly counseling meetings and maintaining treatment agreements [25]. Furthermore, Drainoni et al. reported an increased access to buprenorphine and naloxone as the main advantage of focus group participation, labeling this treatment as a better option than methadone maintenance [25].

Two studies focus on mainly PLWHA experiences with pain management regarding clinical access to and use of prescription opioids, the relationship with pain medications, access to HIV treatment, and the stigma associated with both diseases [26,27]. Claborn et al. explores the clinical staff awareness and perspectives on prescribing opioids as an incentive to retain patients in HIV care was explored [28].

A study conducted at an urban community-based research facility in Baltimore City, MD, described the mixed positive and negative interactions with health care providers regarding chronic pain treatment [26]. Isenberg et al. finds that the participants’ described the relationship favorably as the providers were proactive and willing to address changes in their needs. This willingness manifested through modified medications and therapies when appropriate. In contrast, several other participants stated that they lacked empathy from their provider when pain was inadequately managed, among the many reasons for the negative interactions. These negative experiences led to seeking new providers or mistrusting the medical system [26].

Additionally, this study shows that the participants had a complex relationship with pain medications. For example, participants were unwilling to share their drug use history with their physicians. The main concern from the participants′ point of view was that sharing this information would cause their doctor to withhold the prescription due to assumed drug-seeking behavior or abuse [26]. Another layer of the complex relationship was due to the participants′ fixation on experiences with pain medications as opposed to other therapies. Participants mentioned dissatisfaction with their prescription pain medications. Isenberg et al. concluded that some of the participants were not willing to take pain medications due to their previous illicit drug use. The participants expressed fear that receiving and taking pain medication would be the ultimate effect of drug abuse [26].

Acknowledging challenges for PLWHA and OUD, one study from the inpatient detoxification unit in New York City explores the positive and negative experiences of patients in this vulnerable population related to all aspects of the HIV care continuum, including preferences around HIV/OUD integrated care [27].

Tofighi et al. found that while some participants reported positive experiences with access to testing in the criminal justice system along with the integration of addiction treatment with their HIV care, many patients experienced the stigma of HIV present in society along with many barriers to care [27]. Barriers included insurance limitations, limited awareness of HIV/AIDS Service Administration benefits, and delayed access. PLWHA also reported challenges with adherence following linkage their HIV primary care, citing travel costs and limited access to providers. Others noted a difficulty in obtaining mental health services which led to non-adherence or substance abuse [27].

A secondary analysis from a New England study explored clinical staff awareness and perspectives on prescribing opioids as an incentive to retain patients in HIV care. Healthcare providers included physicians, medical residents, counselors, social workers, clinical supervisors, case managers, nurses, and medical assistants [28].

Claborn et al. show that nine (81%) reported previous experience and/or knowledge of incentivizing treatment with narcotic prescriptions or recommending medical marijuana to retain patients, compared to only one of 12 (8%) SUD providers [28]. None of the other providers included in the study reported previous experience or knowledge of this practice [28]. Both groups of providers identified possible benefits of using prescriptions as incentive for PLWHA including improved appointment compliance, connection to the healthcare system, and patient health outcomes, as well as increased patient motivation [28]. Concerns regarding this type of incentivization were also brought up by providers, citing negative effects on patient/provider relationships, a shifted focus from HIV care to the prescription, manipulation, and enabling SUD in a vulnerable population. Other concerns were related to diversion of the prescribed narcotics if not used by patients [28].

## 4. Discussion

The above studies represent an empirical map of the current literature, which leaves a vast amount of information unknown, this study summarizes all available qualitative studies focused on the role of pharmacists in access to care in PLWHA taking opioid medication in the US. These eight studies represent sparse literature that provides knowledge and information about PLWHA in the US, who are on ART, and used or are currently taking opioid prescriptions, illicit drugs, or use MAT (e.g., buprenorphine/naloxone, methadone); however, these studies are not hypothesis testing. In contrast to quantitative and epidemiological studies that utilize power and statistical analysis to state conclusions, qualitative studies add insights and illuminate the reader on in-depth details of the prevailing socio-cultural issues of HIV and OUD [29,30]. It is important to note that a limited number of the included studies in the systematic review provide substantial information about triangulation of the corpus of data and not focus on how the rigor and trustworthiness were achieved [30,31].

One of the findings of this systematic literature review study is in-depth information about the barriers and facilitators to MAT. The review shows that PLWHA faces obstacles such as transportation issues, housing, discrepancies in knowledge to access the services, required meetings to be maintained in the program agreement. Oldfield et al. reported PLWHA have identified different obstacles to care, such as discrepancies in resources and knowledge among healthcare providers and the health systems [21]. Disparities in resources manifested as differential access to medical versus social services, as well as differential access to HIV- versus OUD-related services [21]. Further, healthcare leaders who participated in this study also reported that there were more considerable resources available to people with HIV with and without OUD than those who have OUD alone [28]. These critical aspects of accessing the healthcare system described by Oldfield et al. highlight the significance of developing an environment where medications are the foundation of treatment for OUD [21]. Haldane’s systematic review finds that better integration of resources and collaborations with providers would be beneficial not only for PLWH, but it will also enhance the service outcome [32].

This review illustrates that the PLWHA and OUD face problematic issues in terms of required participation in weekly counseling meetings and maintaining treatment agreement [22,23,25]. Each study described a variety of counseling interventions, including meetings with social workers or other PLWHA in the form of a support group. Drainoni et al. reported increased access to buprenorphine/naloxone as the main advantage of focus group participation [25]. This study provided evidence that the main obstacle for PLWHA was the obligatory participation in weekly counseling meetings and maintaining treatment agreements [25]. On the other hand, Korthuris et al. reported advantages and disadvantages to buprenorphine at an opioid treatment program where they received individualized substance abuse counseling [22]. In this study, the participants preferred office-based treatment versus other types of treatment due to various advantages such as greater convenience, the development of a trustful relationship, and sympathy from the healthcare providers [22]. This office-based treatment was positively received by all of the participants who believed that this led to a supportive environment for sobriety [22]. Furthermore, Eagen et al. described the vital role played by individualized or group counseling, meetings with the case manager, and other supportive services [23]. These counseling techniques were in addition to pharmacological treatment (i.e., buprenorphine/naloxone) [23]. While most of the participants in the study presented with other comorbidities such as mental health issues or had a chaotic life with multiple incarcerations, counseling proved to be invaluable for them [23]. A systematic literature review echoes the advantages for PLWHA to take buprenorphine/naloxone for OUD and be adherent to the treatment [21].

Only a few studies included in this review highlighted the stigma associated with HIV, OUD, or MAT [21,27]. Although the stigma has been mentioned as an obstacle to care and the policies and practices surrounding privacy, there was limited information about stigma origins and how it impacted the PLWHA’s daily life. New studies in PLWHA and OUD could explore the stigma origins and how to mitigate it.

The ARV regimens available for PLWHA represent the cornerstone and lifesaving; however, the studies included in this systematic review provided limited information on how they impact PLWHA’s lives [21]. It could be argued that numerous studies have shown the importance of adherence to ARV therapy, and this is the reason why it was not captured in these studies. It could be relevant to suggest that further studies are needed to explore the patients’ stories concomitantly using ARV, opioid medications, or MAT.

## 5. Limitations

The current systematic literature review on qualitative studies should be interpreted in light of its limitations. Firstly, grey literature such as non-peer-reviewed reports, conference abstracts, masters or doctoral thesis, and commentary papers have been excluded. Secondly, there were limited number of qualitative studies that addressed the effect of opioid use on ART adherence on the U.S. population. Although numerous qualitative studies were conducted globally, the focus of the current systematic literature review was on the US population. Further systematic literature reviews on qualitative studies could compare and contrast the U.S.’s findings with global ones. Thirdly, the current systematic literature review included only manuscripts published in English. Therefore, literature in other languages would have been missed. Finally, studies conducted on social media were not included in the review due to the novelty of the field and the lack of tools to assess the bias. This suggests that future systematic literature reviews should focus on social media research that could bring more evidence to this subject.

## 6. Conclusions

The present narrative review identified some commonalities including the benefits of being part of a counseling session or receiving counseling for MAT, the difficulties many participants face in receiving pain treatment, delayed access to HIV healthcare providers, and the commencement of ART. Some of the participants expressed a desire to establish relationships with their clinical staff. These studies also present issues such as homelessness, the lack of trust in providers, difficulties in access to MAT, and drug diversion from very different contexts, and therefore, it must be analyzed in the socio-economic context. Furthermore, this empirical literature review of the US qualitative studies could be of interest to policymakers who could develop further programs that address the needs of this heterogeneous population: PLWHA and OUD, PLWHA, and MAT. This empirical literature review showed specific literature gaps in which researchers could conduct studies to explain the role of pharmacists in the integration of care.

## Data Availability

Not applicable.

## Appendix A

MEDLINE Search Strategy: ((Acquired Immunodeficiency Syndrome[Mesh] OR “HIV”[Mesh] OR “acquired immunodeficiency syndrome” OR “human immunodeficiency virus” OR “HIV” OR “AIDS” OR “PLWH” OR “persons living with HIV” OR “Anti-Retroviral Agents”[Mesh] OR “antiretroviral” OR “antiretrovirals” OR “anti-retroviral agent” OR “anti-retroviral agents” OR “anti-retroviral” OR “anti-retrovirals” OR “anti-retroviral therapy”) AND (Opioid-Related Disorders[Mesh] OR “opioid use disorder” OR “OUD” OR “opioid abuse” OR “Methadone”[Mesh] OR “Buprenorphine”[Mesh] OR “Analgesics, Opioid”[Mesh] OR “Opiate Substitution Treatment”[Mesh] OR “opioid agonist therapy” OR “opioid agonist treatment” OR “analgesic” OR “analgesics” OR “opioid” OR “opioids” OR methadone OR buprenorphine)) AND (“Patient Preference”[Mesh] OR “Health Knowledge, Attitudes, Practice”[Mesh] OR “Patient Acceptance of Healthcare”[Mesh] OR “Social Stigma”[Mesh] OR “Shame”[Mesh] OR experience OR experiences OR perception OR perceptions OR shame OR shames OR “social stigma” OR “social stigmas” OR barrier OR barriers OR “quality of life” OR attitude OR attitudes OR belief OR beliefs OR perspective OR perspectives OR “Medication Adherence”[Mesh] OR “Treatment Adherence and Compliance”[Mesh] OR “Treatment Refusal”[Mesh] OR “medication adherence” OR “medication compliance” OR “medication persistence” OR “treatment refusal” OR adherence OR compliance OR noncompliance OR concordance OR “social risk” OR “social risks”).
